# The Pathogenic Roles of IL-22 in Colitis: Its Transcription Regulation by Musculin in T Helper Subsets and Innate Lymphoid Cells

**DOI:** 10.3389/fimmu.2021.758730

**Published:** 2021-12-21

**Authors:** Jun Yan, Jing Yu, Ke Liu, Yijia Liu, Changchuin Mao, Wenda Gao

**Affiliations:** ^1^ State Key Laboratory of Trauma, Burns and Combined Injury, Department of Special War Wound, Research Institute of Surgery, Daping Hospital, Army Medical University, Chongqing, China; ^2^ School of Life Science and Engineering, Southwest Jiaotong University, Chengdu, China; ^3^ Antagen Pharmaceuticals, Boston, MA, United States

**Keywords:** IL-22, Musculin, ILC3, colitis, mucosal immunity, Th17, transcription factor, RORγt

## Abstract

IL-22 plays a crucial role in promoting inflammation, antimicrobial immunity and tissue repair at barrier surfaces. The role of IL-22 in colitis is still controversial: while IL-22 has a protective effect on gut epithelium in acute injuries, it also enhances colitis in a context-dependent manner. Here, we summarize the Yin and Yang of IL-22 in colitis. Particularly, we emphasize the role of innate lymphoid cells (ILCs) in IL-22 production and regulation. A previously underappreciated transcription factor, Musculin (MSC), has been recently identified to be expressed in not only Th17 cells, but also RORγt^+^/Id2^+^ IL-22-producing group 3 ILCs in the gut of naïve mice. We hypothesize that the co-expression and interaction of MSC with the key transcription repressor Id2 in developing lymphoid cells (e.g., in LTi cells) and ILC precursors might fine tune the developmental programs or regulate the plasticity of adaptive Th subset and innate ILCs. The much-elevated expression of IL-22 in MSC-/- ILC3s suggests that MSC may function as: 1) a transcription suppressor for cytokines, particularly for IL-22, and/or 2) a gatekeeper for specific lineages of Th cells and innate ILCs as well. Amelioration of colitis symptoms in MSC-/- mice by IL-22-blocking agent IL-22BP-Fc suggests a counterintuitive pathogenic role of IL-22 in the absence of MSC as a checkpoint. The theory that exuberant production of IL-22 under pathological conditions (e.g., in human inflammatory bowel disease, IBD) may cause epithelial inflammation due to endoplasmic reticulum (ER) stress response is worth further investigation. Rheostatic regulation of IL-22 may be of therapeutic value to restore homeostatic balance and promote intestinal health in human colitis.

## Introduction

Since its discovery more than two decades ago (1), Interleukin-22 (IL-22) has been extensively studied for its roles in maintaining mucosal barrier integrity, antimicrobial defense, cellular proliferation and inflammation. The beneficial and pathogenic roles of IL-22 in various disease settings, and in intestine homeostasis in particular, have been very well summarized in quite a few excellent reviews (2–4). Of note, knowledge on IL-22 cellular sources, receptor signaling, cytokine induction and feedback regulation of its production, as well as IL-22 stimulatory functions on antimicrobial molecules has greatly promoted our understanding of this crucial cytokine in intestinal epithelium regeneration and barrier protection, as well as pathobiology in human diseases, such as rheumatoid arthritis, psoriasis, interstitial lung diseases and colon cancer (5).

On the other hand, the differentiation, plasticity and phenotype maintenance of innate lymphoid cells (ILCs) in IL-22 production is still an uncharted area, awaiting more detailed analysis on the transcription factors (TF) that help define their developmental pathways and phenotypic stability. We propose that pharmacological interventions on specific regulating TFs in ILCs, rather than targeting the secretary products of ILCs, which usually have multifaceted functions (6), could lead to more stable beneficial outcomes in human diseases, maintained by long-lasting ILC populations. For that purpose, herein we first summarize the current knowledge on major TFs involved in the differentiation of both T helper cell subsets and their innate lymphoid counterparts. Then, we will introduce a previously underappreciated transcription repressor, Musculin (MSC), with accumulating evidence for its critical roles in IL-22 regulation, particularly in ILCs. Discoveries in MSC-/- mice support the emerging theory that IL-22 overproduction in colitis underlies intestinal epithelial cell stress response, and call for cautious re-evaluation on IL-22 supplementation therapy for human IBD.

## T Helper Cell Differentiation and Shared TFs in ILCs

Cellular differentiation requires the precise actions of lineage-specifying TFs. In the immune system, naïve CD4+ T helper cells differentiate into distinct lineages with unique cytokine profiles. Besides T helper type 1 (Th1), Th2, Th17 and T regulatory (Treg) cells, with their master TFs being T-bet, GATA-3, RORγt/RORα and Foxp3, respectively, newer subsets such as Th9, T follicular-helper (Tfh), as well as Th22 cells have also been recognized (7–13). Although much has been known about the extracellular cytokine cues on the differentiation of classical T helper subsets (Th1, Th2, Th17 and Treg) (7–9, 14), it remains to be pinpointed, besides those master TFs, what Th-specific intracellular regulators contribute to maintaining the phenotype and function of Th subsets.

While T helper cells are an established realm of adaptive immunity, innate lymphoid cells (ILCs) have recently come into focus (15, 16). ILCs lack antigen specificity, but are particularly enriched at barrier and mucosal surfaces to regulate tissue homeostasis and immune responses. ILC family consists of a variety of developmentally-related innate immune cells, as they all depend on the transcriptional repressor, inhibitor of DNA binding 2 (Id2), as well as on the common cytokine receptor γ-chain for their development (17, 18). Based on their cytokine secretion profiles, ILCs fall into three major groups (15): ILC1s, ILC2s and ILC3s, in parallel with the prominent CD4+ Th1, Th2, and Th17 subsets, and express similar transcription factors and cytokines (19, 20). Namely, ILC1s express T-bet and produce interferon-γ (IFN-γ) (21); ILC2s are enriched with higher levels of GATA-3 and express IL-5, IL-9 and IL-13 (22, 23); ILC3s are featured with the transcription factor RORγt and secrete the Th17 cytokines IL-17 and IL-22 (24). A possible fourth group IL-10-secreting regulatory ILCs (ILCregs) with unique surface markers and transcription signatures has been identified in the intestines of mice and humans (25). ILCregs inhibit ILC1- and ILC3-induced intestinal inflammation in an IL-10-dependent manner, yet unlike Tregs, they are highly enriched in Id3 but do not express Foxp3 (25). The existence of ILCregs is still controversial (26), as other groups suggested that they may originate from IL-10-secreting ILC2s (27, 28).

**Figure 1:**
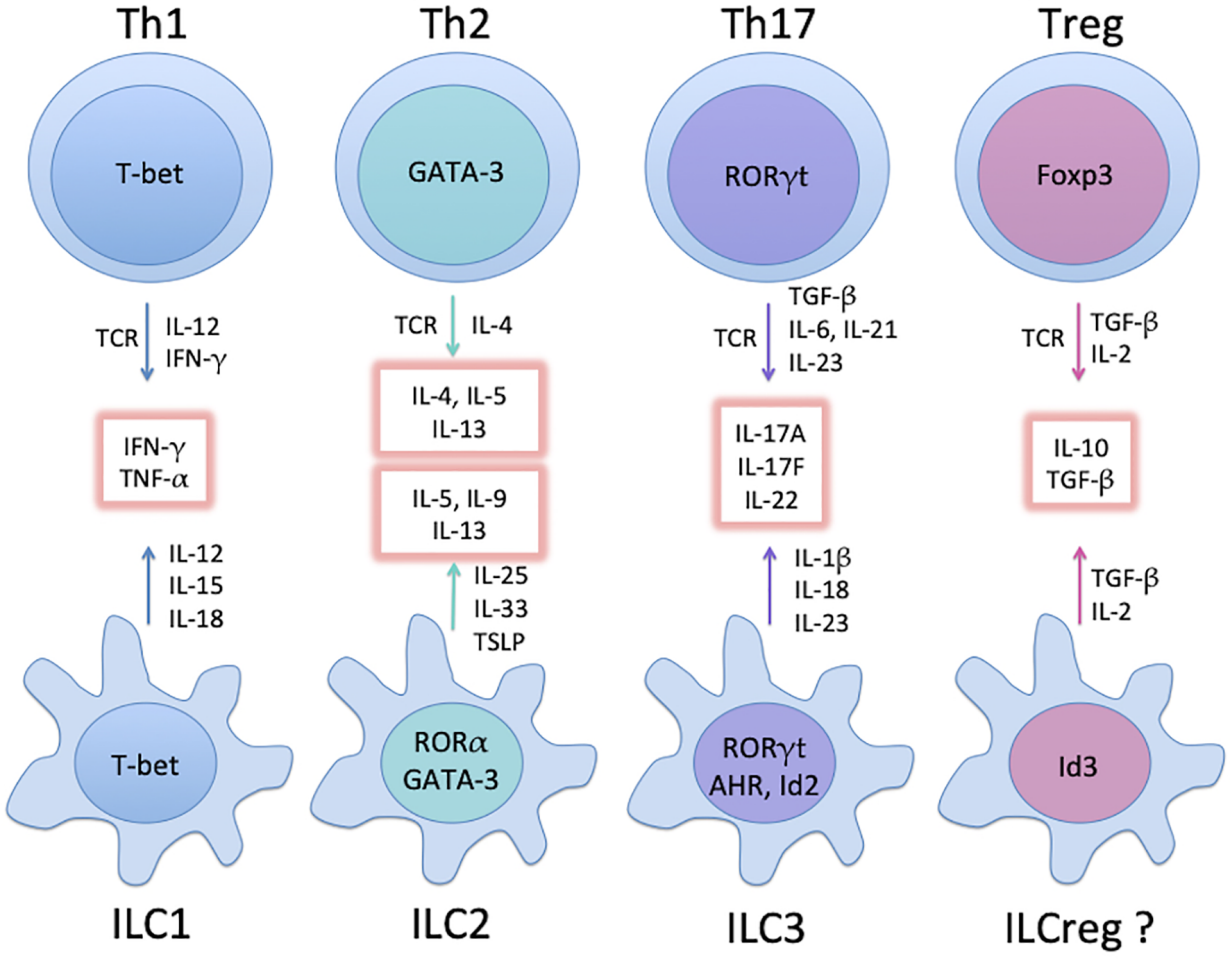
Major groups of Th subsets and ILCs, and their master TFs. Groups of mature ILCs mirror Th subsets with similar cytokine profiles (glowing boxes). Not depicted here are common helper-ILC progenitors (CHILPs) relying on Id2 and GATA-3 for differentiation. While ILC1 and ILC3 develop directly from the CHILPs upon expression of T-bet and RORγt, respectively, further increase in GATA-3 expression leads to the development of the ILC2 precursor (ILC2P) and subsequently ILC2. Note that the existence of ILCreg is still controversial (see text).

RORγt+ group 3 ILCs (ILC3s) are the most heterogeneous ILC population and can be further divided into several subsets: CD4+CD3- LTi (lymphoid tissue inducer) cells that are primarily involved with secondary lymphoid organ formation during embryogenesis, and upon stimulation are able to secrete IL-17A and IL-22 (24). A second subset typically found in mucosal tissues produces IL-22 but not IL-17A, also known as NK22 cells and ILC22s (29). The third subset of ILC3s secretes IFN-γ, IL-17A, and IL-22, and was found to play a pathogenic role in murine innate colitis (30). Thus, the main cytokine produced by ILC3s is IL-22 and some populations of ILC3s also produce IL-17. Akin to their Th counterparts, considerable plasticity exists among ILC groups (16), and trans-differentiation of ILCs could underlie certain diseases (31). The major TFs involved in Th and ILC differentiation are summarized in **Figure 1**.

## Networks of TFs in Lineage Specification and Plasticity of Th Cells and ILCs

As mentioned earlier, “master TFs” were thought to be the main determining factors for specific cell lineages. It is nevertheless becoming more and more appreciated that networks of regulatory TFs collectively contribute to the differentiation program and lineage maintenance. To reveal any additional players other than the “master TFs”, high-throughput microarray and expression profiling technologies along with functional knockout and knockin strategies need to be exploited to delineate the complex regulatory networks of TFs co-expressed within a differentiating or trans-differentiating cell (32, 33).

A good example for such lineage plasticity is between Treg and Th17 cells. While TGF-β induces naïve T cells to differentiate into Tregs, in the presence of TGF-β and IL-6 or IL-21 Treg differentiation is suppressed whereas Th17 development is promoted (14, 34). The reciprocal developmental pathways for Treg vs. Th17 suggest that these two important Th subsets are interrelated and can transform to each other under specific conditions. Indeed, Treg trans-differentiation has been widely observed in autoimmune diseases, such as juvenile arthritis, type 1 diabetes and multiple sclerosis (35), as well as in transplant rejection (36). In an inflammatory microenvironment enriched with IL-6 and IL-23 cytokines, “ex-Tregs” are converted into IL-17+ or IFN-γ+ effectors and are functionally indistinguishable from Th17 or Th1 cells, respectively (37).

Likewise, Th17 cells also demonstrate considerable plasticity and readily downregulate IL-17 secretion and turn into Th1-like cells in autoimmune and chronic inflammatory conditions (38–40). In the tumor microenvironment (TME), tumor-induced Foxp3-negative IL-17A+ Th17 cells decline in number at later time points, concomitant with the increase of “ex-Th17” Foxp3+IL-17A- suppressive tumor-associated Tregs (41). Th17-to-Treg trans-differentiation is fostered by TGF-β and tumor-derived PGE_2_ (41). While TGF-β in conjunction with IL-6 and IL-23 promotes Th17 cell differentiation and IL-17A secretion, the presence of PGE_2_ inhibits this effect and promotes the conversion of Th17 cells into Foxp3+IL-17A- cells (41). Thus, TME-derived extracellular immunosuppressive factors play a major role in reprogramming Th cells (41). But what intracellular factor(s) determining the fate of metastable Foxp3+IL-17A+ cells in the late stage at the fork of trans-differentiation is largely unknown.

Like Th cells, plasticity of ILC subsets in reflection of the tissue microenvironment has been a hot area of intensive studies (16). Uncovering additional TFs driving such trans-differentiation in both Th subsets and ILCs is of great importance in understanding the phenotype stability of these immune cells. Modulating TFs that are associated with this process could have significant therapeutic value for human diseases. This is because that unlike in naive animals for de novo disease induction, in human diseases, lineage specification of pathogenic Th/ILC cells may have already taken place at the time when symptoms are manifested. Thus, the window of opportunity for pharmacological intervention may exist at the stage of lineage maintenance and trans-differentiation. In light of this, we now introduce a novel TF, Musculin, which could be implicated in regulating Th and ILC stability, particularly in connection with IL-22 production in ILC3s.

## Musculin (MSC) as an Emerging TF Fine-Tuning Both Th Cells and ILCs

### MSC in Non-Lymphoid Cells

MSC (also known as MyoR (42), ABF-1 and bHLHa22) is a basic helix-loop-helix transcription repressor that is involved in the regulation of muscle development (42, 43). MSC can bind to an E-box motif (CANNTG) either as a homodimer or as a heterodimer with E2A gene products, where the dimers bind the same E-box sequence as myogenic bHLH/E protein heterodimers, but MSC functions as a potent transcriptional repressor that inhibits myogenesis and E-box-dependent muscle gene activation (44). MSC shows two isoforms (45): a 201 aa protein (MSC 1a) and an equally effective 180 aa short form (MSC 1b), with a repressor domain between aa 158-173. Although early studies indicate that MSC has a muscle-specific, developmental stage-restricted expression profile, more recent studies support the notion that “Musculin” could very well be a misnomer, as its expression is also found in several adult tissues of non-muscle lineage. For instance, MSC is expressed in kidney side population (SP) cells, which are enriched with stem cells, and can regulate their function (46).

### MSC in Lymphocyte Differentiation

In lymphoid compartment, human MSC is known as activated B cell factor-1 (ABF-1). MSC facilitates the formation of germinal center B cells and memory B cells, but inhibits the differentiation of antibody-secreting plasma cells (47). In Hodgkin’s lymphoma, B cell-specific transcription program is interrupted by overexpression of ABF-1 (a.k.a. MSC) and Id2, which antagonize the function of the transcription factor E2A in B cell lineage determination. As a result, expression of B cell specific genes is lost, whereas genes normally not associated with the B cell lineage are upregulated (48).

The expression and regulatory roles of MSC in T cells, especially CD4+ T helper cells, await further clarifications. One group reported that MSC is induced in murine Tfh but not Th17 subset, yet MSC is not required for the differentiation of Tfh cells, nor plays a significant role in antibody production in response to T-dependent antigens (49). This is in contradiction to the finding that MSC is selectively expressed in human Th17, but not Tfh cells, and inhibits Th17 proliferative response to IL-2 (50). Furthermore, MSC is found to be preferentially upregulated in peripherally-induced Tregs (iTregs) than in other Th subsets, and it promotes unidirectional development of iTregs by inhibiting the Th2 differentiation pathway (51). It is likely that confusion in the expression profiles of MSC stems from the various conditions of *in vitro* Th cell differentiation.

To avoid the potentially non-physiological anti-CD3/CD28-stimulated differentiation, in our own work, we generated alloantigen-activated Th subsets whose TCR recognizes α-chain of I-E in the context of I-A^b^. MSC is dramatically upregulated in alloantigen-stimulated murine Th17, marginally in Th1, but not in Th0, Th2, or iTreg subsets (52). APC-derived IL-23 has a salutary role in MSC induction. In the *in vitro* activation system with anti-CD3/CD28 in the absence of antigen-presenting cells (APC), Th17 cells also express more MSC than Th0 and Th1 cells, but only at later time points (6-8 days) (52). Such kinetics suggests that most researchers could have missed MSC in fully developed Th17 cells if they performed Th subset differentiation in the absence of APC in the first 2–4 days (49, 51). In addition, we found that when naïve CD4**+**CD62L^hi^GFP- cells from IL-17A^GFP^ knockin mice were differentiated and FACS-sorted based on GFP intensities, MSC correlates with stronger Th17 phenotypes (higher IL-17A/IL-17F/RORγt/RORα/Id2) (52). Clearly, MSC is a Th17-specific TF in both human (50) and mouse (52) systems.

### MSC in Th Lineage Commitment and Stability: Effects on the Promoters of Master TFs

#### RORC Promoter

A glimpse of transcription regulation on the promoter activities of master TFs may give clues on how MSC may affect Th lineage commitment and phenotype maintenance. For instance, transcription of RORγt in the developing Th17 cells is regulated by E-proteins, and E-box binding sites are essential for RORγt promoter activities (53). TGF-β and IL-6, the cytokines promoting Th17 differentiation (14), sequentially induce the expression of E-proteins and Id3 (53). Id proteins are a family of transcription regulators that bind to E-proteins, interfering with their DNA-binding capacity and transcriptional activities (54–57). Within this family, Id2 and Id3 are the main players in the regulation of E-protein function during lymphoid development (58, 59). Both of these Id proteins, as well as MSC, are expressed in Th17 cells (52, 53). As MSC interacts with Id2 to antagonize the function of E2A (44, 48), it is highly possible that MSC could directly or indirectly participate in Th17 lineage specification through modulating E2A activities on RORγt transcription. Particularly, as MSC is expressed at a later time point during Th17 differentiation (52), MSC is more likely involved in Th17 stability. Future studies using promoter-luciferase reporter assay and comparing MSC+/+ and MSC-/- Th17 cells may reveal the role of MSC in regulating RORγt transcription and Th17 differentiation and stability.

#### FOXP3 Promoter

Similar in Th17 cells, differentiation of Tregs is also controlled by E2A and Id3 (60). Enhanced binding of E2A to the *FOXP3* promoter increases Foxp3 transcription, and Id3 potentiates this process by mitigating the inhibitory effect of GATA-3 at the *FOXP3* promoter (60). Inflammation-induced Treg-specific expression of Id2 reduces Foxp3 expression by blocking E2A binding to its promoter, leading to the induction of Th17-related cytokines and the “ex-Treg Th17” phenotype (61). Thus, it is suggested that Id2 triggered by inflammatory stimuli de-stabilizes Tregs into Th17 cells (61).

Although we were unable to detect MSC expression in natural Tregs (nTregs) or alloantigen-stimulated *in vitro* induced Tregs (iTregs) (52), nTregs stimulated by anti-CD3/CD28 microbeads in the presence of IL-6 do express increased amounts of MSC, concomitant with the expression of IL-17A/F and RORγt/RORα, compared to resting nTregs or nTregs stimulated by anti-CD3/CD28 beads only (**Supplementary Figure 1**). In addition, GFP-labeled nTregs from Foxp3GFP knockin mice when adoptively transferred into RAG-1-/- mice can be recovered after 2 weeks into post-RAG-GFP+ and post-RAG-GFP- populations. Compared with nTregs, the post-RAG-GFP- cells lost Foxp3 expression, but upregulated IL-17A/F, RORγt/RORα and Id2 expression. Most interestingly, while the post-RAG-GFP+ cells had an intermediate presentation of Foxp3 and Th17 signature genes compared with nTregs and post-RAG-GFP- cells, they expressed the highest amount of MSC (hundreds of fold increase) among the three groups of cells (**Supplementary Figure 2**). Thus, MSC triggered by IL-6 or homeostatic proliferation in lymphopenic hosts could de-stabilize Tregs for Th17 programming, possibly through interaction with Id proteins to modulate E2A transcription regulation of master TFs. It would be highly intriguing to test this hypothesis by transferring MSC-/- nTregs from Foxp3GFP×MSC-/- mice into the RAG-1-/- mice, and determine the fate of these nTregs during homeostatic proliferation. These experiments are currently underway.

#### AHR Promoter

Besides the promoters of *RORC* (53) and *FOXP3* (60) that contain E-boxes, a conserved canonical E-box sequence is also present in the promoter of aryl hydrocarbon receptor (AhR) (62). Chromatin immunoprecipitation (ChIP) experiments showed that the E-box motif in the promoter region of *AHR* is specifically associated with Myc and its heterodimeric partner Max (62), both of which are members of the basic helix-loop-helix (bHLH) leucine zipper (LZ) family of transcription factors. Homodimerization of Max or heterodimerization between Max and Myc allows these proteins to bind the E-box (CACGTG) (63). It remains to be determined whether MSC binds the E-box sequence in the *AHR* promoter.

Our own work using Foxp3GFP mice showed that alloantigen-stimulated iTregs isolated by GFP-based FACS sorting from the primary mixed lymphocyte reaction (MLR) expressed none to low levels of MSC and AhR, but their expression levels significantly increased during the secondary MLR, concomitant with dramatic expression of Th17 effector molecule osteopontin (OPN) and greatly diminished expression of Treg-associated CCR7 (64) (**Supplementary Figure 3**). This suggests that co-expression of MSC and AhR underlines the metastability and transition of ex-Tregs into Th17-like effector cells.

As E2A plays a crucial role in orchestrating lymphoid differentiation in early multipotent progenitors (57, 65), factors affecting E2A activities could have significant impact on the genes regulated by E2A. ChIP coupled with high-throughput genome-wide sequencing in human myoblasts revealed that MSC shows widespread binding at tens of thousands of genomic sites. Its consensus recognition motif has the palindromic sequence of “CCAGCTGG” (66), overlapping with the canonical E-box sequence “CAGCTG” (67). Thus, MSC could obstruct E2A binding to the E-box sites in the promoters of the master TFs, as a major mechanism of regulating Th differentiation and stability.

**Figure 2:**
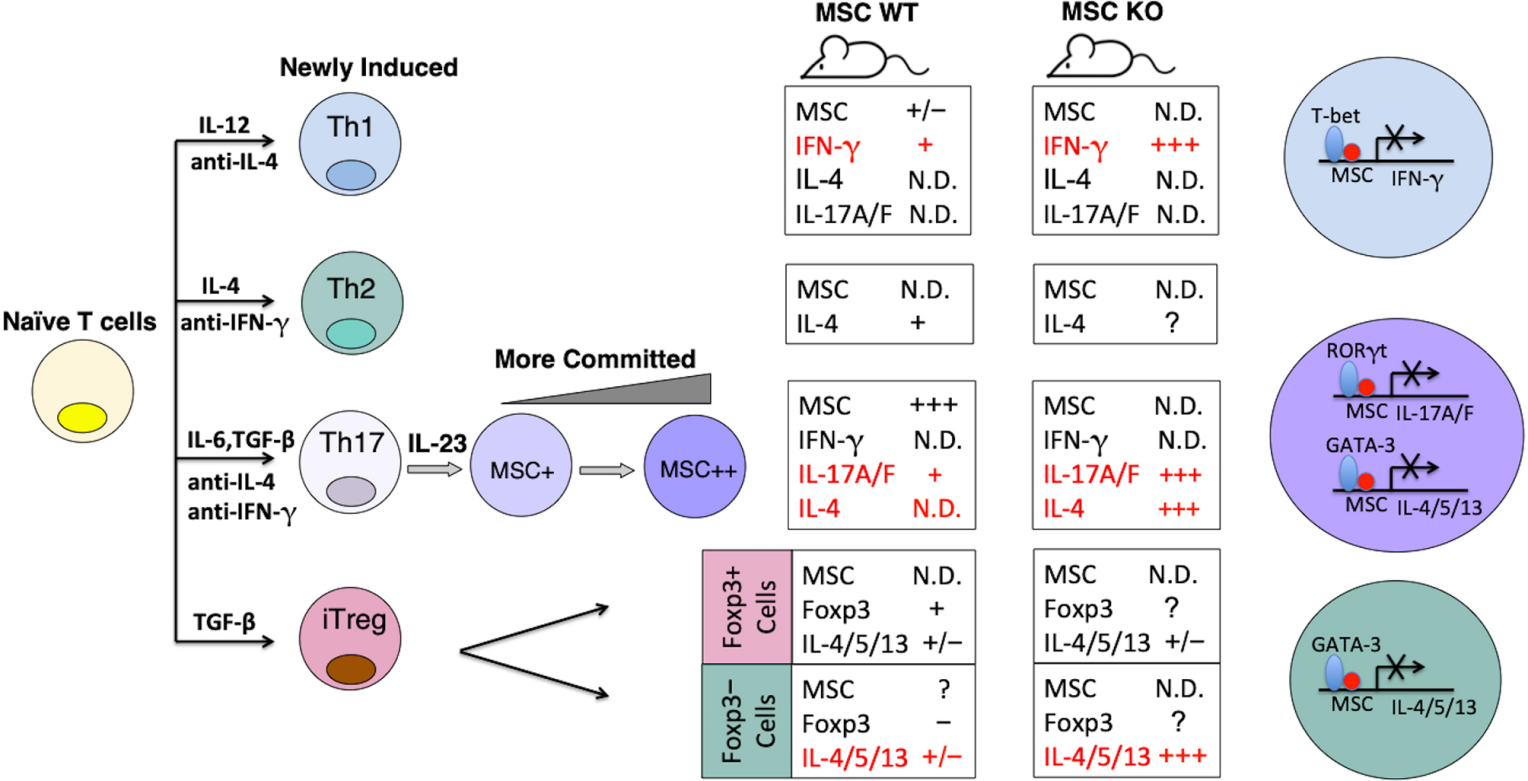
Model of MSC regulation of the differentiation and stability of Th subsets. The expression levels of MSC correlate with stronger Th17 phenotypes. Summaries of gene expression patterns in the boxes correspond to the Th subsets on the left. N.D, not detectable. The major role of MSC in specifying Th differentiation is to suppress E-box-dependent activation of master TFs.

**Figure 3:**
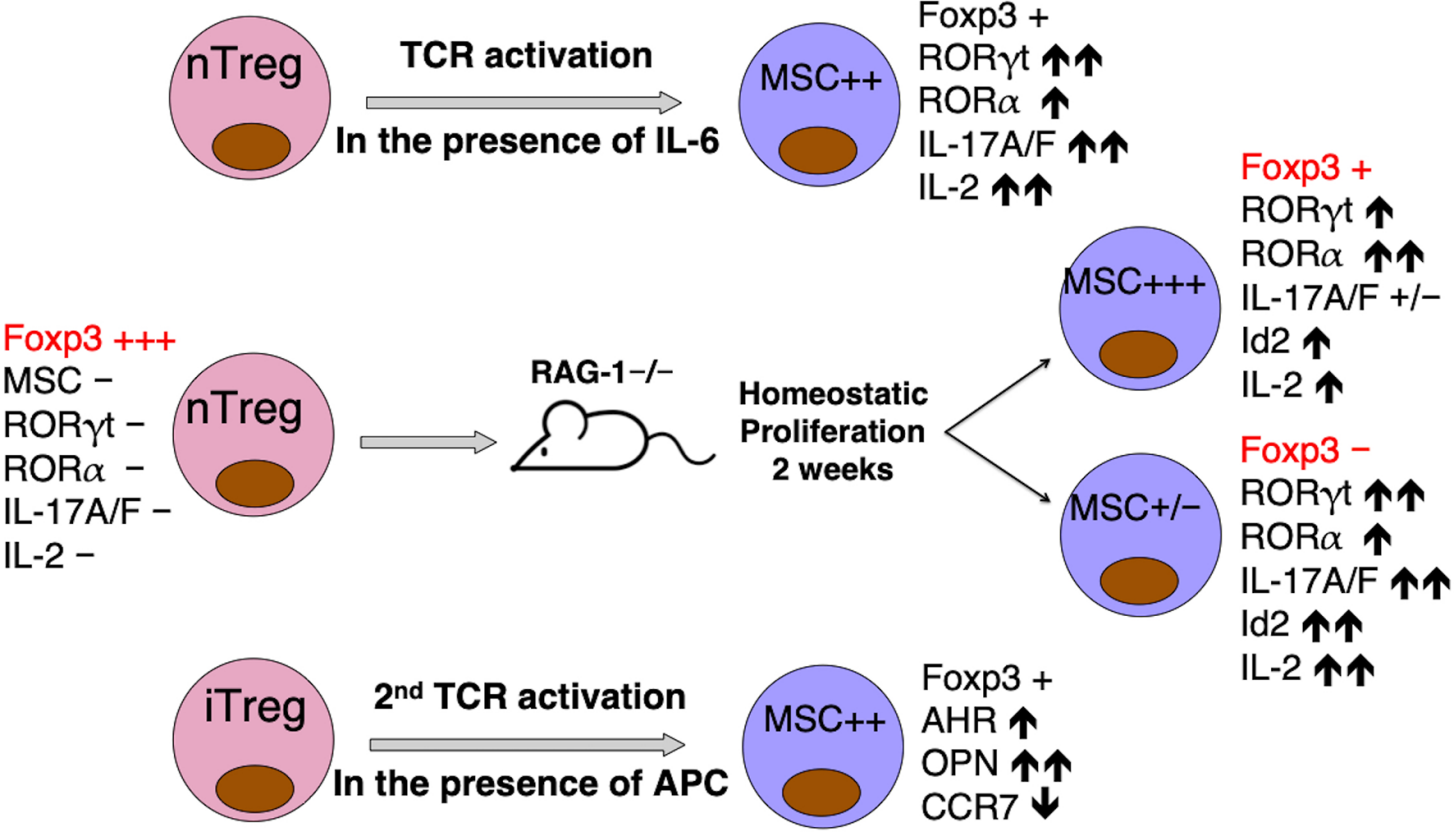
MSC may be responsible for Treg instability and trans-differentiation into Th17 cells. MSC upregulation correlates with the expression of Th17-specific signature molecules, when nTregs are activated in the presence of IL-6 *in vitro* (**Supplementary Figure 1**), or *in vivo* when homeostatic proliferation drives nTregs “parked” in RAG-1-/- mice for 2 weeks to differentiate into two distinct populations, with strong MSC expression marking a metastable Foxp3^int^ population on route to the complete trans-differentiation into a MSC^lo^ Foxp3^neg^ Th17-like population (**Supplementary Figure 2**), or when FACS-sorted alloantigen-activated iTreg are re-stimulated in mixed lymphocyte reaction (**Supplementary Figure 3**). MSC and co-expressing Id2 in these cells may interfere with E2A activation of FOXP3 promoter (61).

Summarizing what is known about MSC expression and function, we propose a model of MSC regulation of the differentiation and/or stability of Th subsets (**Figures 2**, **3**, and **Supplementary Figures 1-4**). MSC is maximally induced in Th17 cells at later time points in the presence of APC-derived IL-23, and marginally induced in Th1 cells, but not in Th0, Th2 or iTreg cells (52). The major role of MSC in specifying Th differentiation is to suppress E-box-dependent activation of master TFs. For instance, it blocks unorthodox expression of GATA-3-mediated Th2 gene IL-4 in Th17 cells (**Supplementary Figure 4**). MSC also suppresses the transcriptional activities of mater TFs in their corresponding host cells, such as T-bet in Th1 and RORγt in Th17 cells, so that these cells are not exhausted. MSC-/- Th1 cells secrete huge amounts of IFN-γ, but are prone to apoptosis (**Supplementary Figure 4**). Note that MSC is not expressed in Foxp3+ iTregs *per se*, and could be involved in Treg trans-differentiation into Th17 cells (**Figure 3**).

Because of the antagonizing effects of cytokines from opposing lymphocyte subsets, caution should be taken in interpreting the data. For instance, Wu et al. proposed that MSC is required for iTreg induction by repressing GATA-3-mediated Th2 programming, as MSC deficiency reduced expression of the master TF Foxp3 in iTreg cells and induced Th2 development even under the conditions promoting for iTreg differentiation (51). Of note, the authors did not separate the Foxp3GFP+ cells from Foxp3GFP- cells during iTreg induction. It is likely that IL-4 boosted by MSC deficiency (51) from Foxp3GFP- cells suppresses Foxp3 expression in the nearby Foxp3GFP+ cells (68) in a paracrine mode. By a similar fashion, Id3 was claimed to be required for Foxp3 induction, as Id3-/- T cells failed to generate iTregs in the presence of TGF-β1 (60). However, when anti-IL-4 was added to the system, induction of iTreg by Id3-/- T cells was restored to the level by Id3+/+ T cells (60). Perhaps more definitive answer to probe for the role of additional TFs in regulating the differentiation and stability of Th subsets and ILCs should come from the knockin system where MSC, or Id3 or other potential factors are expressed in a linked manner with the master TFs, hence cell intrinsic effect of the regulators can be studied on a per cell basis, without the interference from exogenous cytokines.

### Possible Role of MSC in ILC3 Differentiation and Cytokine Secretion

Recently, AhR has been implicated as a master regulator involved in the postnatal maintenance of intestinal RORγt+ ILCs and intraepithelial lymphocytes (69). Various populations of IL-22-producing ILCs all express RORγt and AhR, and all have a shared requirement for IL-23 to produce IL-22 (24, 70). As all the ILC progenitors express Id2 (71) and MSC interacts with Id2 to antagonize the function of E2A (44, 48), MSC could directly or indirectly participate in ILC development through modulating E2A activities on ROR**γ**t and AhR transcription, as discussed above. More studies are needed to reveal the role of MSC in ILCs.

In our own work, we examined Rorc(γt)^+/GFP^ mice whose RORγt^GFP+^ cells are highly enriched in early thymic CD4+ cells and small groups of LTi/Th17/ILCs found in the gut-associated lymphoid tissues (GALT) (72). In Peyer’s patches (PP), memory CD62L^lo^RORγt^GFP+^ cells are either CD4+ or CD4- (52). Compared to CD4+RORγt^GFP−^, both CD4+RORγt^GFP+^ and CD4-RORγt^GFP+^ cells express high levels of MSC, as well as RORγt, RORα and Id2 (52). While CD4+RORγt^GFP+^ LTi/Th17 cells express IL-22 and IL-17A, CD4-RORγt^GFP+^ cells express more IL-22, RORγt and RORα, but no IL-17A (52), suggesting they contain IL-22-secreting ILC3s (a.k.a. NK22 cells or ILC22s). Thus, MSC is uniquely co-expressed with transcription factors RORγt/RORα/Id2 in CD4+IL-17A+ (LTi/Th17) cells, as well as in CD4-IL-17A^−/lo^ populations, the latter of which contains IL-22-producing ILC3s (52). Taken together, MSC is expressed not only in freshly induced Th17 cells, with MSC levels correlated to more committed Th17 phenotypes, but also in RORγt+ IL-22-producing ILC3s in the Peyer’s patches of naïve mice.

Indeed, MSC has a more profound effect on cells from the GALT (such as PP and mesenteric lymph nodes, MLN) than from the spleen and peripheral lymph nodes, which explains why meaningful differences between MSC+/+ and MSC-/- Th subsets are somewhat elusive when naïve cells of spleen or lymph node origin were used for differentiation. For instance, upon Th17 induction by TGF-β1 and IL-6, MACS-enriched CD4+ cells from pooled MLN of MSC-/- mice secreted more IL-22 than the wild type counterparts. Under the same condition though, there was no difference between CD4+ T cells from the spleens of MSC-/- and MSC+/+ mice (52). Moreover, the selective production is more dramatic for IL-22 than for IL-17. In line with this, PP cells from MSC-/- mice express much higher levels of IL-22 than MSC+/+ PP cells, and this difference is greatly amplified during colitis (52). All these results suggest that the elusive effect of MSC is on: 1) a special population of IL-22-secreting cells in the GALT, most likely ILC3s, and/or 2) synthesis and secretion of IL-22 cytokine *per se*. More experiments are needed to distinguish whether MSC is a negative gatekeeper for ILC3 developmental pathway, and/or a checkpoint TF controlling IL-22 synthesis.

## Discussion: The Yin and Yang of IL-22 in Colitis

### Beneficial Effects of IL-22: on Epithelial Cell Regeneration

In order to maintain a functioning barrier, all the intestinal epithelial cells are replenished within days by continuously proliferating stem cells residing in the crypt region. Given the role of IL-22 in supporting intestinal LGR5+ stem cell mediated epithelial regeneration (73), as well as its stimulation on the synthesis of antimicrobial peptides (AMPs), it is no surprise that the prevailing view is for IL-22 promoting gastrointestinal health (73–80). For instance, IL-22 plays a beneficial role in acute intestinal infections where rapid repair of colonic epithelium is required, such as infection caused by *Citrobacter rodentium* (74, 81). Likewise, IL-22 facilitates the restitution of epithelium upon inducing acute colonic injury in dextran sulfate sodium (DSS)-induced murine colitis (75). In cancer chemotherapy, dose-limiting side effects are mainly caused by toxicity in the mucosal surfaces as a result of their high mitotic index. For example, chemotherapy drug methotrexate can induce acute mucositis featured by damage in the epithelium of small intestines, in which IL-22 plays an important restorative role in this self-resolving condition (76). Taken together, the primary insult in these examples is epithelial disruption, where IL-22 drives epithelial cell proliferation and restitution, restoring epithelial barrier. These data have promoted the paradigm of IL-22 being clinically beneficial to repair epithelial damage in human inflammatory bowel disease (IBD), and has led to a clinical trial (NCT02749630), evaluating the role of recombinant IL-22-Fc (Efmarodocokin alfa) in patients with active IBD.

### Pathogenic Effects of IL-22: on Immune Dysregulation and ER Stress Response

However, the key difference between animal models of colitis and human IBD is that, the former usually results from acute insults on the epithelial barrier with self-limiting tissue injury that can quickly resolve upon withdrawal of the insult, whereas the latter is characterized by chronic immune-mediated inflammatory reactions. While IL-22 definitely has a protective role on gut epithelium (82, 83), it also promotes colitis in a context-dependent manner (30, 84), especially when immune components are involved. For instance, in an adoptive transfer colitis model when Treg-depleted memory/effector CD4+CD45RB^lo^ T cells were transferred into RAG-1-/- mice, IL-22 derived from these memory/effector T cells is pathogenic, whereas IL-22 is protective when CD4+CD45RB^hi^ naïve T cells were transferred (84). Note that in this chronic colitis model, transfer of Treg-depleted CD4+CD45RB^lo^ T cells into RAG-1-/- mice was characterized by a Th1<Th17 cytokine signature in the colon tissues, whereas transfer of CD4+CD45RB^hi^ naïve T cells demonstrated a Th1>Th17 cytokine signature (84). In an innate colitis model with anti-CD40 administration in RAG-1-/- mice, IL-22-neutralizing antibody significantly reduced the colon pathology, colitis scores and weight loss triggered by anti-CD40 injection, whereas IL-22 plasmid administration exacerbated colitis with infiltration of inflammatory cells and mucosal hyperplasia (30). Interestingly, in that model, the only source of IL-22 in the gut of RAG-1-/- mice is RORγt+ ILC3s, which are required for colitogenesis (30). Thus, it seems that the pathogenic role of IL-22 could be associated with ILC3s and RORγt+ Th17 cells (e.g., through its soluble factor, IL17A, for chemotaxis of immune cells).

Our own work also supports the notion that heightened IL-22 expression by ILC3s when MSC was deficient has an aggravating effect in DSS-induced murine colitis (52, 85). While the wild type mice survived DSS-induced colitis, MSC-/- mice showed increased production of pro-inflammatory cytokines (e.g., IL-22, IL-6, TNF-α) in the gut, with more infiltrating immune cells in colon tissues, reduced body weight, and earlier onset of death. The epithelial injury in the colon tissues was much severer in MSC-/- mice than in the wild type counterpart during colitis, with the counterintuitive observation of higher concentrations of IL-22 in the supernatants of ex vivo cultured colon tissues from the MSC-/- group than from the wild type animals (52, 85). Surprisingly, administration of IL-22BP-Fc, an Fc fusion protein containing the antagonizing IL-22 binding protein (IL-22BP), ameliorated the colitis symptoms in MSC-/- mice (52). This is the first report demonstrating a beneficial effect of IL-22BP in colitis model, where other studies showed the opposite (86, 87). Similar to our finding, in an acute polymicrobial sepsis model, IL-22 blockade by IL-22BP-Fc was also shown to be protective against bacteria spread and organ failure (88). Clearly, a balanced production of IL-22BP is context dependent for intestinal homeostasis.

In addition to the animal data, recent human studies also challenge the dogma of IL-22 being beneficial for colitis, especially during chronic inflammation, instead of in acute, self-limiting mucosal injury (89, 90). First, IBD is not an acute inflammatory disease. Environmental and genetic factors, as well as gut microbiome, cause stable changes of epithelium functions, which have been considered as the major etiology of IBD (91, 92). Second, a humanized monoclonal antibody (Mab) Risankizumab that specifically blocks IL-23, which triggers IL-22 production, demonstrated promising results in a Phase II clinical study in IBD (89). In patients with moderate to severe Crohn’s disease (CD) who had failed prior treatment with TNF-α blockade, another human IL-23-blocking Mab (MEDI2070) also showed clinical improvement in a Phase 2A study (93). Of note, in that study, patients with higher baseline serum IL-22 levels were more responsive to MEDI2070 (93). More detailed mechanistic studies using colonic epithelial organoids and tissue transcriptome analysis in a large datasets of CD patients revealed that elevated IL-22 orchestrates a pathological endoplasmic reticulum (ER) stress response, amplified by IL-17A (90) which is a cytokine normally co-produced with IL-22 by ILC3s (94).

It should be brought to attention that, in IBD patients, persistently elevated IL-22 levels could alter the baseline of the inflammatory responses in the colonic epithelium. IL-22 induces TNF-α, inducible nitric oxide synthetase (iNos2) and apoptosis-inducing caspase 12 and STING (90), which can trigger ER stress. ER stress is caused by overwhelmed protein synthesis under adverse stimuli such as infection and non-physiological cellular insults, resulting in the accumulation of aggregated mis-folded proteins that are potentially toxic to the cells (95). To mitigate these harmful effects, cells develop a highly conserved protective process called unfolded protein response (UPR). IL-22 significantly stimulates proteins involved in regulating UPR in colonic epithelium (90). For example, IL-22-induced iNos2 is a trigger as well as a downstream effector of the ER stress response (96). In addition, iNos2 causes DNA damage associated colon cancer in chronic colitis (97). IL-22 also significantly upregulates TLR-4 and its signaling adapter MyD88 in colonoids (90). Engagement of this pattern recognition pathway likewise induces ER stress in colonic epithelial stem cells (98). IL-22-induced pro-inflammatory molecule STING can increase small intestinal epithelial apoptosis by inducing Type I Interferon production (99). Thus, the role of IL-22 in the context of chronic inflammation during IBD needs to be carefully re-evaluated, and the supplementation of exogenous IL-22, especially in an Fc fusion with long serum half-life, to patients with active colitis should be re-considered with extreme caution.

### IL-22BP: A Natural Antagonist to Re-Establish Intestinal Homeostasis

Nature always has checks and balances. A classical example of a naturally-existing cytokine antagonist is interleukin-1 receptor antagonist (IL-1RA). IL-1RA is not an alternatively spliced form of the extracellular domain of any receptor, but is encoded by a separate gene. It binds non-productively to interleukin-1 receptor (IL-1R) and functions as a molecular sink to quench the activities of IL-1α and IL-1β, modulating a variety of IL-1-related immune and inflammatory responses (100). In a very similar fashion, IL-22 has its own nemesis: IL-22 binding protein (IL-22BP, a.k.a. IL22RA2) (101, 102). IL-22BP is a soluble receptor, 231 amino acids in length with 34% sequence homology to IL-22R1 (101, 103), but binds IL-22 with more than 1,000-fold higher affinity than the membrane form IL-22R1 (104) and inhibits IL-22 biological functions by sequestration from its targets (105).

IL-22BP is produced by epithelial cells, eosinophils, macrophages and dendritic cells (106). While IL-22BP expression is significantly increased during IBD, because of the elevated numbers of IL-22BP-producing eosinophils in human gut (86), it should be kept in mind that such increase could be cause or consequence of colonic inflammation, in relation to the surge of IL-22. More relevant to the context of this review, although many reports suggest a detrimental effect of IL-22BP at blocking the protective effects of IL-22 in acute colitis (75, 86, 87), the role of IL-22BP in chronic IBD, in addition to its beneficial effect on blocking IL-22-mediated tumorigenesis (87) and in acute polymicrobial sepsis (88), warrants further investigation. First, species and model differences have produced some contradictory observations. IL-22BP is highly expressed by immature DC in the normal colon but significantly reduced during acute inflammation in DSS-induced murine colitis (75, 107, 108). In contrast to findings in mice, in patients with IBD increased expression of intestinal IL-22BP was rather observed (86, 87). Second, unlike in mice, human IL-22BP has three functionally different isoforms, which are generated by alternative splicing (109). Isoform 1 is very poorly secreted and fails to antagonize IL-22 signaling. More recently, isoform 1 was found to be largely retained in ER and functions as an intracellular activator of the UPR response (110). Isoform 2 is the only isoform that is secreted in large amounts when myeloid cells are activated by retinoic acid or Toll-like receptor 2. Isoform 3 is the most widely distributed, but its affinity to IL-22 is 27-fold lower than that of isoform 2 (109). Thus, human IL-22BP isoforms play distinct temporal and spatial roles fine-tuning the functions of IL-22.

Regardless of its expression profiles and rheostatic regulation on IL-22, IL-22BP is considered a potential therapeutic target. For example, IL-22BP-Fc reduces the severity of cutaneous pathology and inflammation in the mouse model of psoriasis (111). Anti-IL-22BP may have therapeutic value for influenza infection, as a pro-IL-22 environment was shown to alleviate pulmonary inflammation in IL-22BP-knockout mice (IL-22RA2−/−) during H1N1 (PR8/34 H1N1) infection and protected the lung by promoting tight junction formation (112). It needs to be actively pursued whether IL-22BP has therapeutic value for human IBD, in lieu of IL-22-induced colonic epithelial ER stress and completely attenuated chronic colitis in IL-22-/- mice or by anti-IL-22 neutralization (90). Testing IL-22BP-Fc as a therapeutic venue in this model and in clinical trials should be a logical next step.

## Hypothesis: Role of MSC in IL-22-Mediated Pathology

Our own work showed for the first time that exuberant production of IL-22 by GALT cells in the absence of a transcription suppressor, MSC, aggravates colitis even in the acute DSS model (52, 85). We also found that during colitis induction by DSS, IL-22RA1 expression in colonic epithelial cells was greatly enhanced in MSC-/- mice compared with MSC+/+ mice, as detected by immunohistochemistry staining (52). Thus, MSC deficiency enforces IL-22 signaling by upregulating both the cytokine and its receptor. Reversal of colitis symptoms by antagonistic IL-22BP-Fc suggested that signaling through supraphysiological amounts of IL-22 and its receptor IL-22RA1 in the gut is detrimental, and could possibly induce colonic epithelial ER stress and consequent apoptosis (90).

**Figure 4:**
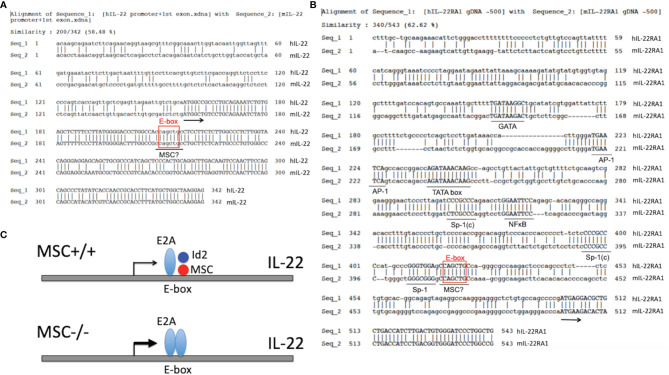
Possible negative regulation of IL-22 and IL-22RA1 by MSC. **(A)** Alignment of the promoter and the first exon regions of mouse and human IL-22 genes. E2A binding site is boxed, which overlaps with a potential MSC binding motif. Arrow indicates the start of translation. **(B)** Upstream 500-bp 5’ flanking regions of the mouse and human IL-22RA1 genes are aligned to show the putative TATA-boxes and transcription factor binding sites (underlined). Sp-1(c) indicates complementary to a SP-1 binding consensus. E2A binding site is boxed, which overlaps with a potential MSC binding motif. Arrow indicates the start of translation. **(C)** Diagram of IL-22 transcription regulation by MSC. An E-box motif in the first exon of IL-22 attracts E2A binding and enhancement of IL-22 transcription. This effect is counteracted by MSC : Id2 heterodimer recognizing a similar motif overlapping with the E-box. In the absence of MSC, this negative brake is released, and hence more E2A is bound to the E-box leading to the enhanced transcription and translation of IL-22. A similar scenario may also apply to the transcription regulation of IL-22RA1 by MSC.

Interestingly, we spotted a highly conserved sequence “CCAGCTGC” at +53 nt position downstream of the ATG start codon of both human and mouse IL-22 genes (**Figure 4A**), which contains an E-box sequence in the center and is only one nt different from the consensus MSC binding motif “CCAGCTGG” (66). Similarly, we also noticed a highly conserved DNA segment containing an E-box sequence “CAGCTG” next to the SP-1 binding site at around -80 nt position upstream of the ATG start codon of both human and mouse IL-22RA1 genes (113) (**Figure 4B**). Thus, both IL-22 and IL-22RA1 could be yet another set of target genes affected by MSC regulation of E-box activities. A diagram of MSC regulating IL-22 production is proposed (**Figure 4C**). More studies using gene promoter luciferase reporter assay and EMSA assay are needed to probe whether the potential regulation by MSC on the expression of cytokines and their receptors is a universal phenomenon, or only specific to certain cytokine families, or to cytokines from certain cell subsets.

**Figure 5:**
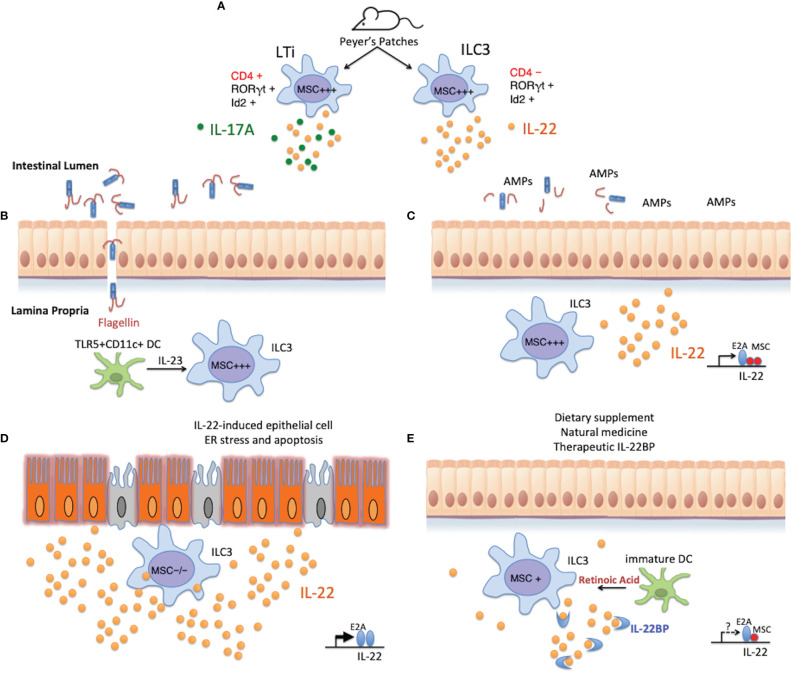
Potential role of MSC in regulating intestinal homeostasis. **(A)** MSC is highly expressed in LTi cells and ILC3s isolated from the Peyer’s patches of naïve mice, and could regulate the development or cytokine secretion of these cells in a similar fashion as in Th subsets where MSC is induced. **(B)** When the intestinal mucosal barrier is compromised, microbes enter the intestinal lamina propria through the damaged epithelium. TLR5+CD11c+ lamina propria dendritic cells detect the presence of flagellated bacteria *via* the flagellin receptor TLR5, and rapidly secrete IL-23 to alarm the ILC3s to promptly produce IL-22. **(C)** MSC+ ILC3s produce copious amounts of IL-22, which stimulates epithelial cells to secrete antimicrobial peptides (AMPs) and their regeneration for barrier restitution. This process is negatively regulated by MSC in these cells. **(D)** Under pathological conditions, such as in IBD or in MSC-/- mice during acute DSS-induced gut insult, exuberant production of supraphysiological amounts of IL-22, unchecked by the transcription repressor MSC, triggers ER stress in the epithelium that leads to cell apoptosis and chronic inflammation. **(E)** Re-establishing intestinal homeostasis with natural remedies (such as introducing probiotics) or pharmaceutical intervention on the IL-22:IL-22BP balance is possible, only when knowledge on the physiological regulation of the producing cells, as well as the mechanism of disease induction by IL-22 is adequately obtained. Pharmacological targeting of MSC (e.g., by RA) could lead to more stable beneficial outcomes in IBD, maintained by long-lasting Th and ILC populations.

Besides setting brakes on the expression of cytokine(s) and/or cytokine receptor(s), it is highly plausible that MSC contributes to the differentiation and lineage stability of Th subsets and ILCs. Alternatively, MSC could be involved in stem cell longevity and renewal activity. Regardless of the mechanism, MSC-/- mice provide an unprecedented opportunity to study the potential pathogenic effect of IL-22 in disease models. Through understanding the expression and regulation of MSC *per se*, we might be able to develop pharmacological agents that act on MSC and/or its expressing cells to re-establish long-lasting intestinal homeostasis (**Figure 5**).

## Future Perspectives

The dichotomy of Yin and Yang is the basic concept of Chinese philosophy – viewing the world as not just opposing powers, but more of dynamic ever-changing elements that can transform into each other under different conditions. IL-22 and IL-22BP are such good examples, i.e., not only IL-22 is a multifaceted cytokine, having diverse beneficial and pathological effects, but also IL-22BP joins the convoluted network of checks and balances. Thus, simple strategy of adding (e.g., IL-22BP-Fc) or removing (e.g., anti-IL-22) elements from the system may be mission-impossible to achieve transformation and ultimate re-establishment of homeostasis. Even worse, such simple strategy when fueled by incomplete understanding of the disease models and mechanisms, such as supplementing IL-22-Fc to IBD patients with chronic inflammation, could have dire consequences.

What we have learned from our studies on transcription factors in general, and MSC in particular, in Th/ILC differentiation and cytokine regulation is that, key regulators more often than not have many downstream targets. Future medicine should consider adopting a strategy to target multiple pathways, perhaps by the assistance of AI and machine learning. For example, the vitamin A metabolite retinoic acid (RA) has been shown to promote Treg but inhibit Th17 differentiation (114). RA also stimulates γδ T cells and innate lymphoid cells to produce IL-22 and attenuates intestinal inflammation (115). In addition, we found that IL-6-stimulated MSC expression is boosted by IL-23 but suppressed by RA (**Supplementary Figure 5**). Thus, the attenuating effects of RA on colitis may be linked to its suppression on MSC to release the brake for the expression of physiological levels of IL-22 for cytoprotection. Nevertheless, RA also significantly induces IL-22BP by immature monocyte-derived DC in the gut to antagonize the role of IL-22 (108). A thorough investigation on the multi-effects of RA on wide targets from IL-22, IL-22BP, transcription factors such as MSC, to Treg/Th17/ILC differentiation will gain us a holistic view on how the Yin and Yang forces are working in health and disease, and guide future treatment strategies. Perhaps in another 10 years, AI programs will become powerful enough to assist the analysis from single chemical drugs to multiple modalities as complex as traditional Chinese medicine, which has been tested for thousands of years to be effective in restoration of balance in certain diseases, such as Indigo Naturalis or Qingdai for IBD, where part of the mechanisms seems to involve AHR activation and IL-22 induction (116, 117). But then again in colitis or IBD, how much IL-22 is just good, and how much is too much? Can we achieve a rheostatic regulation of IL-22 to restore homeostasis? Such questions can only be answered when the roles of all the molecular and cellular players for IL-22 regulation, including MSC, are sufficiently understood.

## Data Availability Statement

The original contributions presented in the study are included in the article/**Supplementary Material**. Further inquiries can be directed to the corresponding author.

## Ethics Statement

The animal study was reviewed and approved by IACUC of Army Medical University.

## Author Contributions

JYa, JYu, KL, and YL collected data in own work in colitis studies. CM contributed with critical reagents used in colitis model, and WG designed research conceptually and wrote the manuscript. All authors have read and agreed to the published version of the manuscript.

## Funding

This research was funded by Natural Science Foundation of Chongqing (cstc 2018jcyjAX0258), and Science and Technology Innovation Enhancement Project of Army Medical University (2019XYY22).

## Conflict of Interest

Authors CM and WG are employed by Antagen Pharmaceuticals, Inc.

The remaining authors declare that the research was conducted in the absence of any commercial or financial relationships that could be construed as a potential conflict of interest.

## Publisher’s Note

All claims expressed in this article are solely those of the authors and do not necessarily represent those of their affiliated organizations, or those of the publisher, the editors and the reviewers. Any product that may be evaluated in this article, or claim that may be made by its manufacturer, is not guaranteed or endorsed by the publisher.
