# Supplementary material for: The Pathogenic Roles of IL-22 in Colitis: Its Transcription Regulation by Musculin in T Helper Subsets and Innate Lymphoid Cells
Source: Front Immunol. 2021 Dec 21;12:758730. doi: 10.3389/fimmu.2021.758730 (PMC8724035; doi:10.3389/fimmu.2021.758730)
Supplement: Supplementary file 1 [file DataSheet_1.pdf]

Supplementary Material

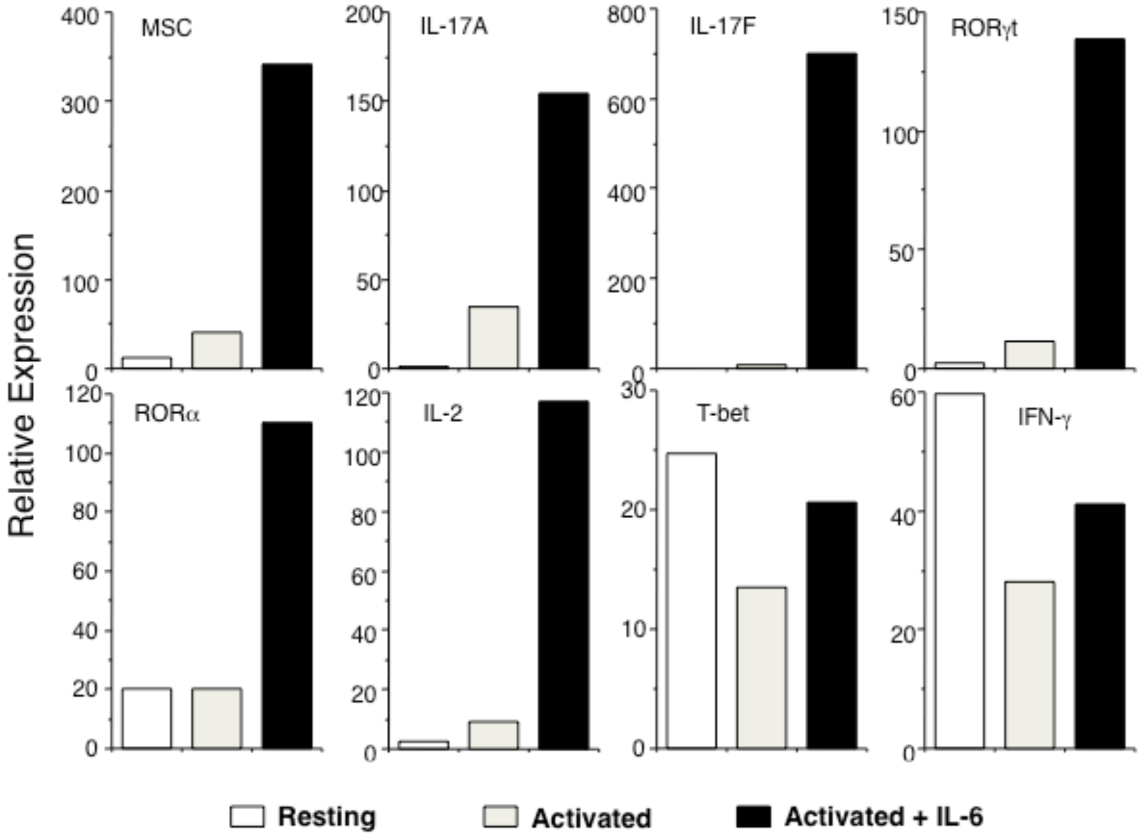

Supplementary Figure 1. MSC is expressed during Treg trans-differentiation into Th17 cells in vitro. FACS-sorted GFP+ nTregs from Foxp3GFP knockin mice were stimulated with anti-CD3/CD28 microbeads (Miltenyi Biotec) supplemented with IL-2 (20 ng/mL), and with or without IL-6 (20 ng/mL) for 3 days. qRT-PCR was performed for gene expression profiling in freshly sorted GFP+ nTregs (Resting), nTregs stimulated with the microbeads (Activated), and nTregs stimulated with the microbeads in the presence of IL-6 (Activated + IL-6). nTregs activated in the presence of IL-6 upregulate MSC, as well as signature genes for Th17, but not for Th1. Data of duplicated wells are presented. $P<0.05$ between Resting and Activated + IL-6 for MSC, IL-17A, IL-17F, RORγt, RORα and IL-2 (Student's t-test). Not significant between Resting and Activated + IL-6 for T-bet and IFN-γ.

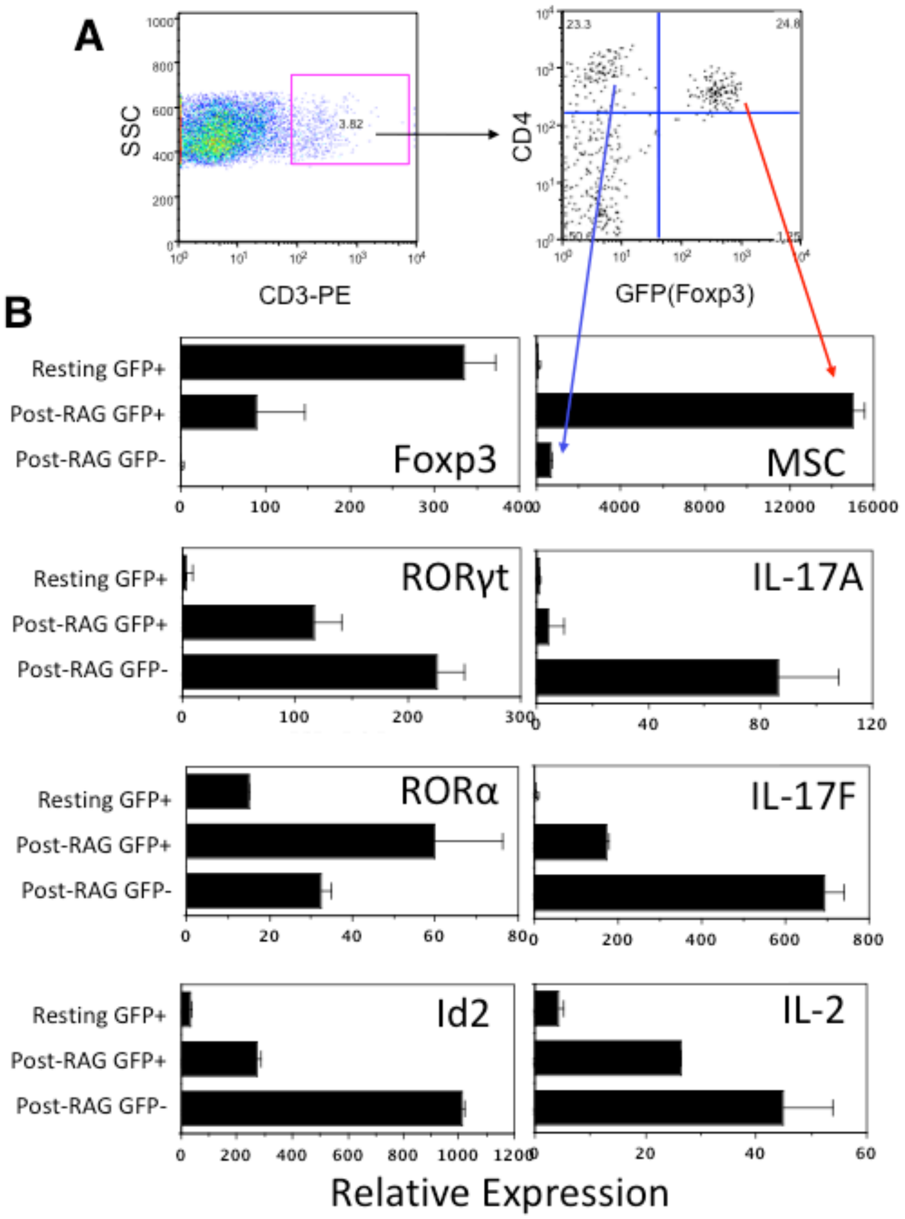

Supplementary Figure 2. MSC is induced during Treg trans-differentiation into Th17 cells in vivo. (**A**). FACS-sorted GFP+ nTregs from Foxp3GFP knockin mice were adoptively transferred into RAG-/- mice for 2 weeks. After homeostatic proliferation, CD3+ cells were recovered and FACS-sorted into CD4+GFP+ (red arrow) and CD4+GFP- (blue arrow) populations. (**B**). qRT-PCR was performed for gene expression profiling in freshly sorted GFP+ nTregs (Resting GFP+), post-RAG CD4+GFP+ and CD4+GFP- cells. MSC is highly expressed in post-RAG GFP+ cells, before their complete trans-differentiation into Th17 cells. Data of duplicated wells are presented. $P<0.05$ between Resting GFP+ and Post-RAG GFP- for Foxp3, RORγt, Id2, IL-17A, IL-17F, and IL-2. $P<0.05$ between Resting GFP+ and Post-RAG GFP+ for MSC and RORα (Student's t-test).

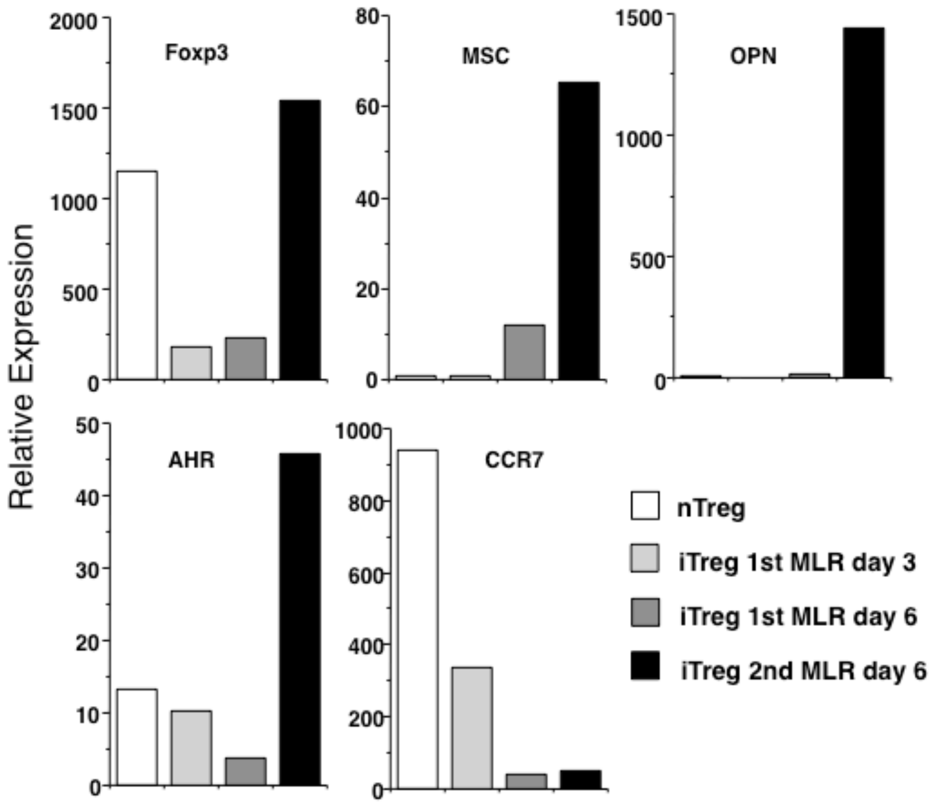

Supplementary Figure 3. MSC is induced in alloantigen-stimulated iTregs during secondary mixed lymphocyte reaction (MLR) and could underlie their unstable phenotype. FACS-sorted naïve CD4+CD62L+GFP- cells from Foxp3GFP knockin mice (C57BL/6 background, $H2^b$) were co-cultured with MACS-enriched B cells from DBA2 mice ($H\text{-}2^d$), in the presence of TGF-β1 (5 ng/mL). Induced CD4+GFP+ iTregs were FACS-sorted from the primary MLR on day 3 and day 6. Sorted iTregs from the day 6 culture of the primary MLR were again co-cultured with MACS-enriched B cells from DBA2 mice for the secondary MLR in the presence of TGF-β1 (5 ng/mL). CD4+GFP+ iTregs from the day 6 culture of the secondary MLR were FACS-sorted. qRT-PCR was performed for gene expression profiling in freshly sorted GFP+ nTregs and alloantigen-stimulated iTregs from the primary and secondary MLR at various time points. Although Foxp3 expression in iTregs was intensified during the secondary MLR, the levels of MSC, OPN and AHR were significantly increased with concomitant decrease of CCR7. Data of duplicated wells are presented. $P<0.05$ between nTreg and iTreg 2nd MLR day 6 for MSC, OPN, AHR and CCR7 (Student's t-test).

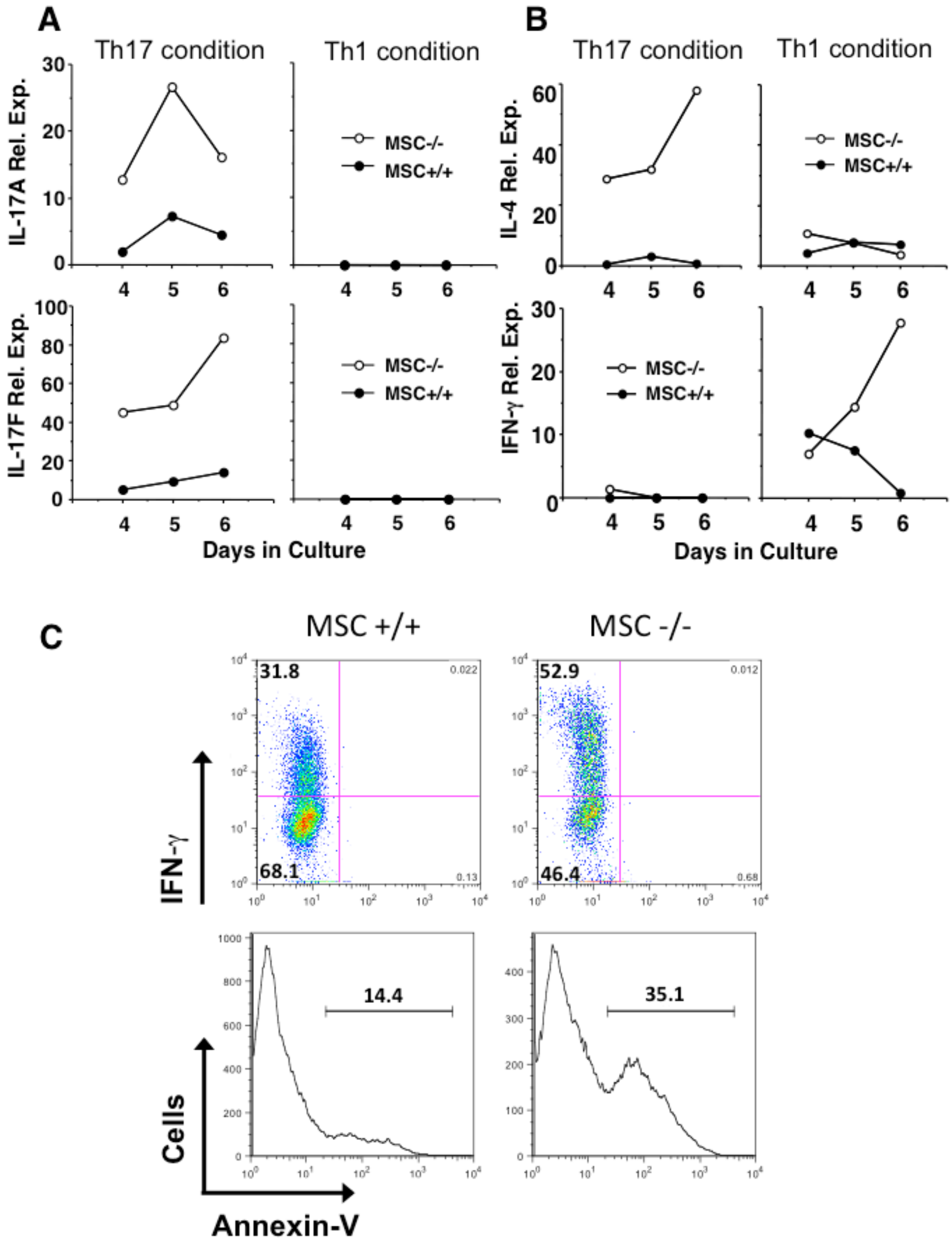

Supplementary Figure 4. MSC negatively regulates cytokine expression in Th17 and Th1 cells. FACS-sorted naïve CD4+CD39-CD25- T cells from the mesenteric lymph nodes of MSC+/+ and MSC-/- mice were differentiated under Th1 and Th17 conditions with anti-CD3/CD28 stimulation. mRNA was extracted at days 4, 5, and 6 in culture for qRT-PCR analysis of IL-17A/F, IL-4 and IFN-γ under both conditions. PCR data from duplicate wells are representative of three similar experiments. MSC suppresses IL-17A/F in Th17 cells (**A**), IFN-γ in Th1 cells as well as unorthodox expression of IL-4 in Th17 cells (**B**). Intracellular cytokine staining showed that MSC-/- Th1 cells produce greater levels of IFN-γ, but are prone to apoptosis as indicated by Annexin-V staining (**C**).

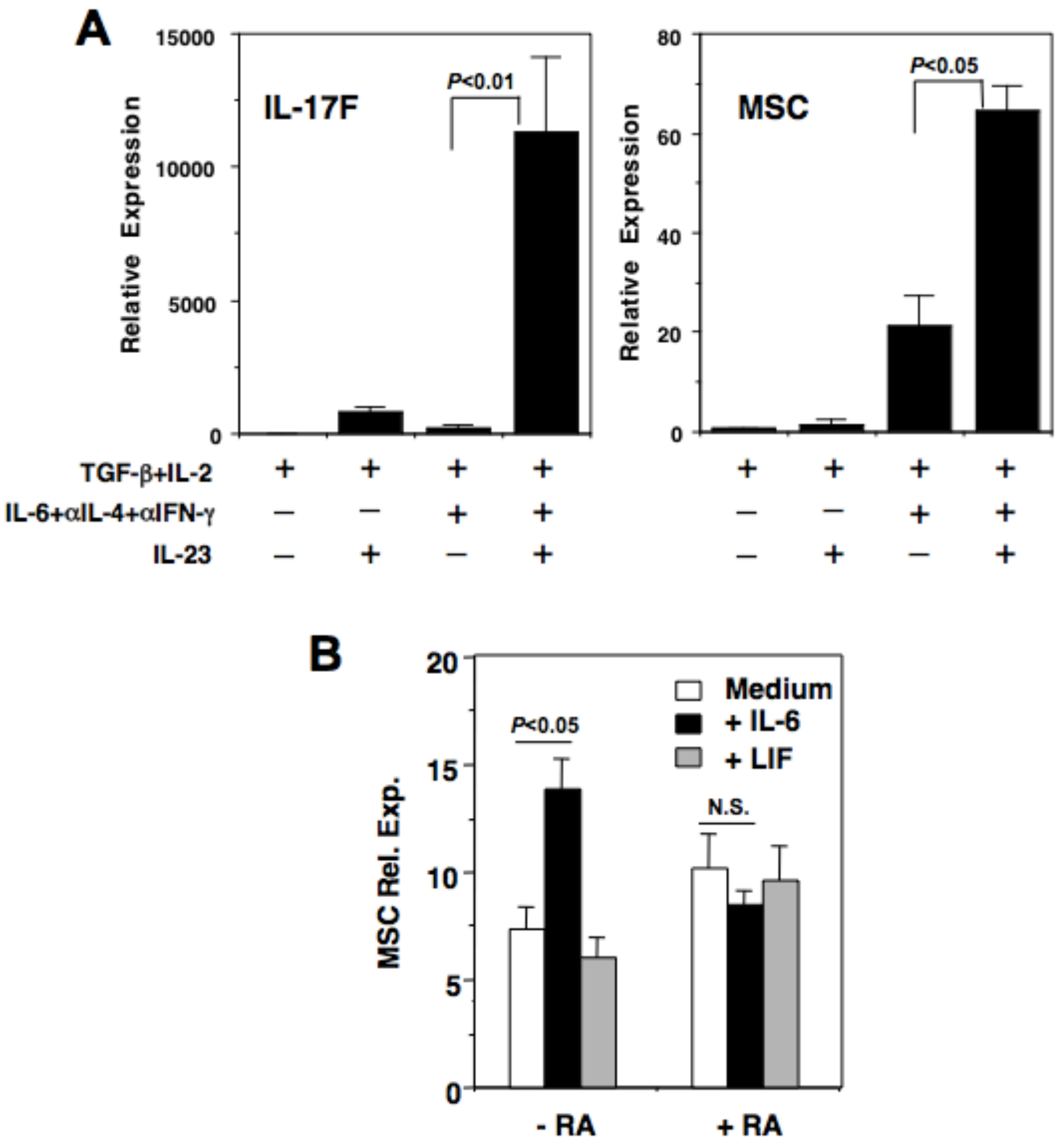

Supplementary Figure 5. IL-6-stimulated MSC expression is boosted by IL-23 but suppressed by retinoic acid (RA) under Th17 differentiation condition. (**A**). FACS-sorted naïve CD4+CD62L+GFP- T cells from Foxp3GFP mice were stimulated with plate-bound anti-CD3 (10 μg/mL) and soluble anti-CD28 (1.0 μg/mL) in various combinations of TGF-β (5 ng/mL), IL-2 (20 ng/mL), IL-6 (20 ng/mL), anti-IFN-γ (10 μg/mL), anti-IL-4 (10 μg/mL) and IL-23 (20 ng/mL). (**B**). FACS-sorted GFP+ nTregs from Foxp3GFP knockin mice were stimulated with anti-CD3/CD28 microbeads (Miltenyi Biotec) supplemented with IL-2 (20 ng/mL), and either IL-6 (20 ng/mL) or LIF (20 ng/mL) for 3 days. RA was added at 100 nM. All cytokines were from R&D Systems, and cytokine antibodies were from eBiosciences. IL-17F and MSC expression were measured by qRT-PCR. Mean ± SD is shown. N.S.: not significant (Student's t-test).
